# Comparison of sequence variants in transcriptomic control regions across 17 mouse genomes

**DOI:** 10.1093/database/bau020

**Published:** 2014-03-18

**Authors:** Cao Nguyen, Abdul Baten, Grant Morahan

**Affiliations:** ^1^Centre for Diabetes Research, The Western Australian Institute for Medical Research, Western Australia, Australia, ^2^Centre of Medical Research, University of Western Australia, Perth, Western Australia, Australia and ^3^Southern Cross Plant Science, Southern Cross University, Lismore, New South Wales, Australia

## Abstract

The laboratory mouse is the most widely used mammalian model organism in biomedical research, so a thorough annotation of functional variation in the mouse genome would be of significant value. In this study, we compared sequence variation in a comprehensive list of functional elements (e.g. promoters, enhancers and CTCF binding sites) across 17 inbred mouse strains. Sequences were derived for ∼300 000 functional elements experimentally identified by the mouse ENCODE project as regulating gene expression in 19 different tissue sources. We aligned sequences for each predicted *cis*-regulatory element to genomes of 17 mouse strains. This yielded a database comprising ∼5 million aligned sequences, allowing interrogation of sequence variation of functional elements for each of the 19 tissues/cell types in commonly used mouse strains. We also developed an online tool to visualize the genome around each predicted *cis*-regulatory element in each tissue context and which allows efficient comparison of variation between any two sets of strains. This will be particularly useful in the context of the Collaborative Cross (CC), which was conceived as a powerful new systems genetics resource to accelerate gene discovery. Comprising a large number of inbred strains derived from eight genetically diverse founders, the CC offers rapid mapping and identification of genes that mediate complex traits. We show that, among the 17 sequenced strains, the set of CC founder strains captures the most variability in the ENCODE elements, further emphasizing the value of this resource.

**Database URL:** www.sysgen.org/ecco

## Introduction

The laboratory mouse is the most widely used mammalian model organism for biomedical research owing to the many advantages it provides. Genetic discoveries based on investigation of the mouse allow insights into human traits because of the degree of conservation between the mouse and human genomes. With the assembly of the (almost) complete genome of the C57BL/6J strain, the ability to relate sequence to function was significantly enhanced (1, 2). This has enabled genetic screens in mice to be performed on an unprecedented scale (3), facilitated the creation of a set of null alleles for all genes (4, 5) and accelerated the definition of mouse sequence diversity (6, 7). Despite this great progress, the molecular basis for much morphological, physiological, biochemical and behavioral variation in laboratory mice remains largely unknown (8–10). We still lack a significant amount of knowledge regarding the molecular basis of the majority of genetically influenced phenotypes, from fully or partly penetrant Mendelian effects (11, 12) and nonadditive effects (12), to the quasi-infinitesimal genetic architecture that underlies many quantitative traits (13). Furthermore, most of the functional sequences in the mouse genome have yet to be found; in particular, *cis*-regulatory sequences are still poorly annotated, while most *trans*-regulatory loci have not yet been defined.

To aid in the discovery of regulatory sequences, comparative genomics is a powerful tool (14). However, this method cannot resolve the temporal–spatial functions of such sequences. Recently, chromatin immunoprecipitation sequencing was applied to identify *cis*-regulatory elements in the genomes of several organisms including humans, *Drosophila melanogaster* and *Caenorhabditis elegans* (15–17). The ENCODE consortium is integrating multiple technologies and approaches in a collective effort to discover and define the functional elements encoded in the human genome, including genes, transcripts and transcriptional regulatory regions, together with their attendant chromatin states and DNA methylation patterns (15). Using the same experimental approach, Shen *et al.* (18) produced a map of nearly 300 000 murine *cis*-regulatory sequences that represent active promoters, enhancers and CTCF-binding sites experimentally determined in a set of 19 diverse tissues and cell types. In our database, each of the *cis*-regulatory elements is mapped to a locus in chromosomes of the mouse genomes as defined by the ENCODE project. This provided a comprehensive resource for the annotation of functional elements in the mammalian genome and the study of regulatory mechanisms for tissue-specific gene expression.

Variation in such elements would contribute to differences in gene expression, and ultimately to differences in various phenotypic traits. Conventional approaches for identifying genetic contribution to variable traits use diverse gene mapping strategies that generally are limited by power, speed and precision. The Collaborative Cross (CC), a large set of recombinant inbred strains, was conceived as a powerful new systems genetics resource that could accelerate discovery (19). Among the many advantages it offers is the rapid mapping and identification of genes that mediate complex traits, especially those that can only be determined *in vivo*. A decade in planning and production in Australia, the USA and Israel (19–22), the CC is now reaching maturity (23).

Recently, the sequences of genomes from 17 widely used mouse strains were obtained using next-generation sequencing (24). These included the eight founder strains of the CC (i.e. A/J, C57BL/6NJ, 129S1/SvImJ, NOD, NZO, CAST/EiJ, PWK/PhJ and WSB/EiJ). Collectively, the sequences of these 17 strains allow study of genetic variation in the most commonly used strains of mice.

The availability of these *de novo* genome assemblies allowed us to analyze variation in the context of the CC. In particular, we aimed to make a comprehensive comparison of genetic variation in all of the 300 000 ENCODE genomic features by integrating these elements with the mouse genome sequences. We provide a database comprising the aligned sequences of the predicted *cis*-regulatory elements in each of 19 tissues and cell types. Our online database and search tool can provide visualization of the genome around each predicted element in each tissue context. A powerful application of this database will be comparing variation in ENCODE elements shared differentially between responder and nonresponder founder haplotypes because this can allow rapid identification of causal variants in candidate genes mapped using the CC. Similar web tools are available, for example, web-quantitative trait loci (QTL) (25), eQTL Viewer (26) and Genevar (27), but they are designed for conventional approaches such as gene expression and/or for analyzing human genome.

## Methods

### Data sources

The *de novo* genome assemblies of 17 mouse strains (24) were downloaded from the Sanger Institute's Web site (http://www.sanger.ac.uk/resources/mouse/genomes/). The mouse ENCODE project [(http://chromosome.sdsc.edu/mouse/; cf (18)] provided data for genomic localizations of RNA polymerase II (polII), the insulator-binding protein CCCTC-binding factor (CTCF) and three chromatin modification marks, histone H3 lysine 4 trimethylation (H3K4me3), histone H3 lysine 4 monomethylation (H3K4me1) and H3 lysine 27 acetylation (H3K27ac), in 13 adult tissues, 4 embryonic tissues and 2 primary cell lines.

### Integration of functional and sequence data sets

A window of *k* base pair (max *k *= 50) either side of each *cis*-regulatory element was taken from the mouse genome reference NCBI Build 37 (UCSC mm9 database) and was aligned against the 17 mouse strains by BLAST search (28). We stored the aligned sequences resulting from BLAST into a MySQL database and developed an online tool to allow interactive visualization and comparison of the variations among the 17 sequenced strains. We used the following notations in our study:

#### Total single-nucleotide polymorphisms in a cis-regulatory element

The total number of single-nucleotide polymorphisms (SNPs) counted in the surrounding *k* base pair window.

#### Variable regulatory elements

These contain at least one SNP in the surrounding *k* base pair window. If there was at least one sequence variation around an element in any strains of a group then the group is considered *variable* at the element.

#### Invariable elements

These elements have no SNPs in the surrounding *k* base pair window. *A group* of strains is an *invariable group* for that element if all sequences of the group are invariable at that element.

**Figure 1.**
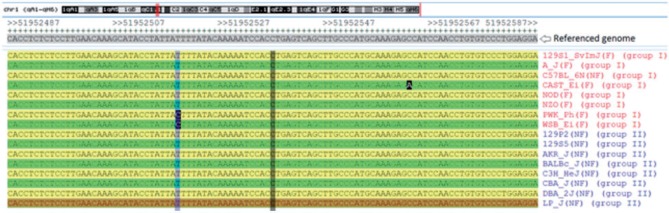
An example for the notations defined in our study. The aligned sequences at chr1: 51 952 487–51 952 587 around an *hek4me3* element in *liver* are shown. Of the total two SNPs (highlighted in black) around the selected element, both were found in the configured group I (red) and neither was found in the configured group II (blue). Group I is considered as variable at this element because there are three sequence variations associated with strains A/J, CAST/Ei, PWK/Ph and WSB/Ei. In contrast, group II is invariable because all the aligned sequences of the group are identical.

Figure 1 shows an example of the above notations. Our web-based tool is written in HTML5/Java script, MySQL and PHP. The database we constructed, termed the Encode CC Omnibus (ECCO), is freely available for user access (www.sysgen.org/ecco).

## Results

### Sequence variations in ENCODE elements

The sequences deriving from *cis*-regulatory elements (polII, CTCF, H3K4me3, H3K4me1 and H3K27ac) for 17 mouse strains in 19 tissues and cell lines were extracted from the mouse reference genome. Using BLAST search against the other sequenced genomes, the relevant elements were selected for each strain. All elements were then aligned to the reference genome. The resulting ECCO database contains ∼5 million aligned sequences of *cis*-regulatory elements (polII, CTCF, H3K4me3, H3K4me1 and H3K27ac) for 17 mouse strains in 19 tissues/cell lines. All sequences are available for download via a button embedded in the Web site.

**Figure 2.**
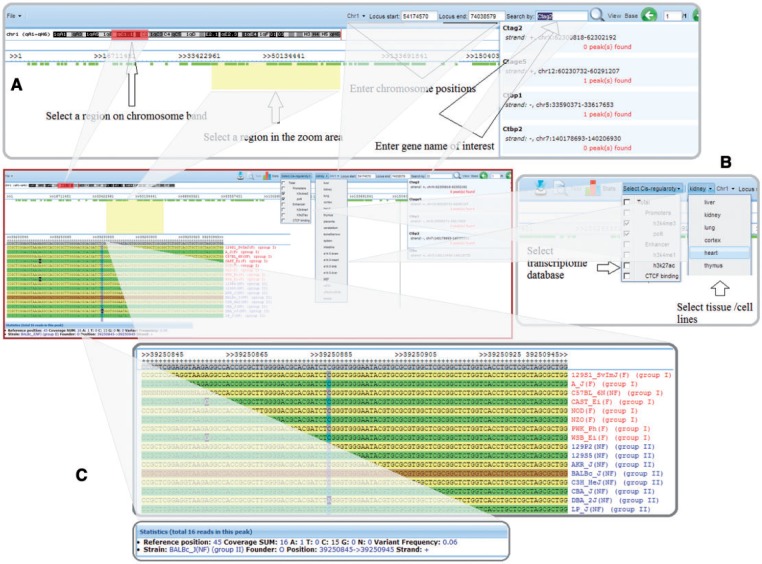
Visualization of *cis*-regulatory elements with sequence variations between the CC founder strains and the set of nine nonfounder strains. (**A**) Users can select a region of interest by four different methods: (i) selecting a region on a chromosome band, (ii) entering chromosome positions, (iii) inputting a gene name in the search box or (iv) highlighting a region in the zoom area; (**B**) Users can select any one or more of the *cis*-regulatory element types and tissue/cell lines for visualization; and (**C**) Strains' genomic sequences are aligned, showing SNPs and statistics.

The SNPs and sequence variations in *k* base pair windows around any of the predicted *cis*-regulatory elements between the CC founder strains or between any of the 17 sequenced strains can be efficiently compared and visualized, as shown in Figure 2. Users can visualize sequence variations in a location of interest either by searching by gene name or by selecting a specific chromosome region. Users can also select the aligned database based on tissue/cell lines and/or *cis*-regulatory elements. The default *k* base pair window is set at 50, but users can specify other values ranging from 1 to 50 for this parameter.

### Sequence variations around *cis*-regulatory elements

Next, we examined the total sequence variations and invariable elements: how many of the predicted elements were variable and invariable, respectively? For each of the aligned sequences in each strain, we determined the number of SNPs, the number of sequence variations in each element (variable elements) and the number of invariable elements.

**Figure 3.**
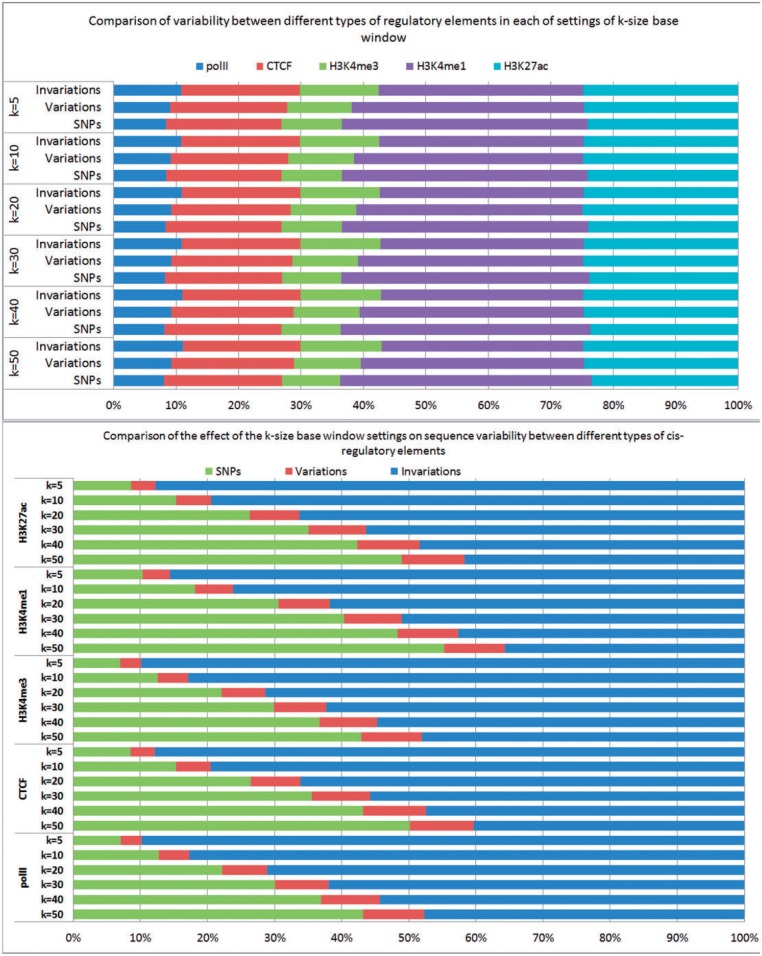
The average number of SNPs, variable *cis*-elements and invariable *cis*-elements in five studied *cis*-regulatory elements for the 19 tissues and cell lines over 17 sequenced strains, for each of settings of *k =* 50, 40, 30, 20, 10 and 5 bp windows, respectively.

Figure 3 shows the results of these statistics in all studied *cis*-regulatory elements for the 19 tissues and cell lines over 17 sequenced strains, for each of settings of *k = *50, 40, 30, 20, 10 and 5 bp windows, respectively. For example, at *k* = 50 bp window, the average number of SNPs per strain for each type of *cis*-regulatory element is ∼567 000, ranging from on average 229 000 (polII) to 667 000 (H3K4me1). Consequently, the total predicted variable elements range from an average 48 000 (polII) to 184 000 (H3K4me1) elements. The full data including number of SNPs, number of variable elements, number of invariable elements for each strain in each studied *cis*-regulatory elements is available at Supplementary Table S1.

### Validation of CC founder strains as source of functional genetic variation

**Figure 4.**
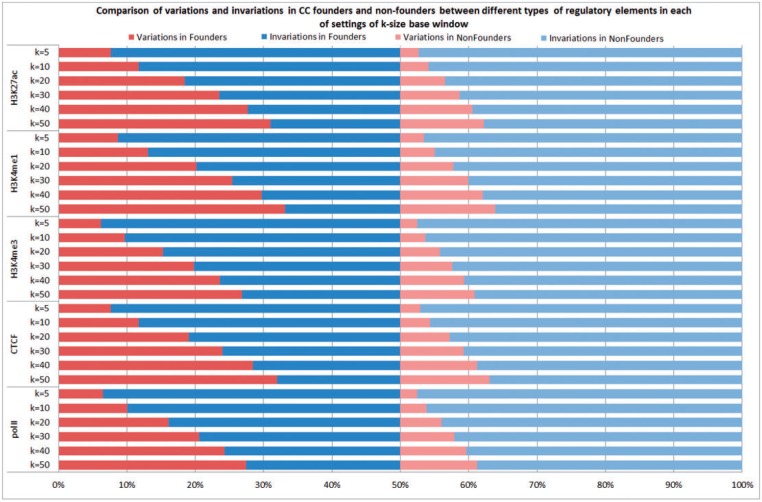
Comparison of variations and invariations in CC founders and nonfounders between different types of regulatory elements in each of settings of k-size base window.

Next, we compared the sequence variations around the predicted *cis*-regulatory elements that were different among the CC founder strains with the other strains (for convenience, referred to below as nonfounder strains). For each group, we counted how many times the group was variable or invariable at each of the *cis*-regulatory elements with *k* base pair windows of 50, 40, 30, 20, 10 and 5, respectively. Note that the definition of variable was liberal: a group at a predicted *cis*-regulatory element was considered as variable if at least one strain had at least one sequence variation in the specified *k* base pair windows. Among the eight founder strains, we found that at *k* = 50 the group was variable at a significantly higher number of elements than were invariable. In contrast, the proportion of variable elements was significantly smaller in the set of nine nonfounder strains. This observation was consistent for all five types of regulatory elements (polII, CTCF, H3K4me3, H3K4me1 and H3K27ac). In addition, we found that in each of the settings of *k =* 50, 40, 30, 20, 10 and 5, the variable elements in founder strains were consistently higher than those in nonfounder strains, suggesting the CC mice would provide a rich source of functional genetic variations (see Figure 4 and Supplementary Table S2 for more details).

### Identification of useful polymorphisms

**Figure 5.**
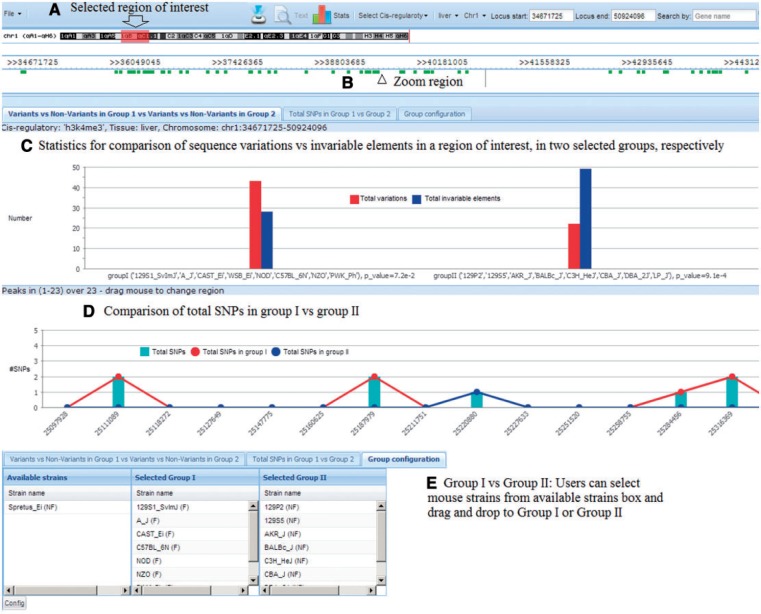
Tool to identify useful polymorphisms. (**A**) Users select region of interest; (**B**) Click ‘View’ to zoom into the region; (**C**) Statistics show the difference between total variations and nonvariations in each group; (**D**) Statistics show the total SNPs in each group and (**E**) Users can select mouse strains for each group.

Another application of our database is to identify SNPs, which could provide useful polymorphisms for further characterization of CC strains, or to select specific strains or even outbred mice [such as the Diversity Outcross; (29)] having particular genotypes at elements of interest for further study. To identify all candidate SNPs for a particular QTL, the database of founder strains' genome sequences can compare responder against nonresponder haplotypes. This allows identification of variable sequences that could be investigated further. These SNPs and microsatellites would be useful to increase map resolution by testing strains with recombinant haplotypes, or to identify individual outcross mice with particular alleles. These could then be tested to confirm and refine locations of QTLs. Users can select regions or genes of interest and configure the groups of responders and nonresponders from the 17 sequenced mouse strains. Our database provides two statistical tools for this comparison: a proportional *z*-test to see the significance level of any difference between variations and nonvariations within a group of strains, and a bar chart for visualization of total SNPs in each group of strains (Figure 5). The *z*-test *P*-value is visible at the bottom of each column as well as when mouse is over the bars in the chart.

### Molecular basis of transcriptional phenotypes

Systems genetics is a powerful technology to analyze effects of genome-wide genetic variants on transcriptome-wide variation in gene expression (30). Here we combined our resource database with the systems genetics approach for systematic investigation of transcriptional phenotypes. Gene expression data from liver, kidney, lung, cortex, cerebellum and spleen were analyzed from a large panel of isogenic recombinant inbred strains of BXD mice [(www.genenetwork.org, cf (28)]. The BXD family is a collection of recombinant inbred strains that were created by successive inbreeding of progeny generated from matings of C57BL/6J and DBA/2J mice. Using WebQTL (25), we extracted highly expressed genes (mean expression >10) likely to have variable *cis*-regulatory elements within 1 MB. These eQTL had peak likelihood ratio statistics (LRS) over 40.

Coordinates for each of these eQTLs were input to our database to further examine sequence variations in *cis*-regulatory elements. Table 1 shows the number of suggestive QTLs with at least one variable element in any of the five *cis*-regulatory elements in the tissues studied. These candidate eQTLs regulate expression of genes that have been associated with traits that are worth investigating in further studies. For example, our analysis showed that *Prp19*, a gene essential for cell survival and DNA repair (31) and linked with tumorigenesis (32), is highly expressed in liver (mean expression at 10.95, max LRS at 129), lung (11.02, 157), kidney (11.03, 95) and cerebellum (11.6, 85.9) and is associated with variable H3K4me1 and H3K27ac elements. The full list of candidate QTLs including gene names, tissue, gene expression variation, the LRS and the number of variable, the total *cis*-regulatory elements and the total variable elements is available in Supplementary Table S3.

**Table 1.** Number of genes associated with transcriptional phenotypes found by webQTL and the ECCO database using the default *k=*50 bp window

Tissue	Genes	Mean expr	Max LRS	Total elements					Total variable elements				
				polII	CTCF	H3K4me3	H3K4me1	H3K27ac	polII	CTCF	H3K4me3	H3K4me1	H3K27ac
Liver	28	11.33 (10.02–15.66)	66.11 (41.5–129)	29	15	23	52	81	12	6	12	25	39
Kidney	59	11.44 (10.07–15.33)	66 (40.1–174)	57	83	62	208	199	24	24	26	70	60
Lung	85	10.89 (10.03–12.95)	70.2 (40.3–196)	64	92	88	331	318	31	29	28	120	115
Cortex	33	11.4 (10.03–14.89)	70.07 (43.3–131)	30	33	22	153	45	13	18	11	63	15
Cerebellum	11	11.92 (10.13–14.31)	60.2 (41.5–89.4)	10	10	13	33	13	6	2	9	21	7
Spleen	35	10.81 (10.01–13.17)	68.01 (41.7–182)	22	23	30	63	78	10	7	13	32	28

Mean expr: the mean gene expression (normalized log2 expression values); Max LRS: maximum likelihood ratio statistics (LOD values / 4.61).

## Discussion

A large fraction of *cis*-regulatory elements act in a cell-type specific manner and are involved in regulating tissue-specific gene expression (18). The ECCO database incorporates flexible statistical methods to analyze ∼300 000 *cis*-regulatory elements in 19 different tissues and cell lines. We were able to show that CC founder strains contained significantly more sequence variations in *cis*-regulatory elements in any of the studied tissues and cell lines than did nonfounder strains. Our web-based tool is also available, so researchers may select specific strains for further investigation and comparison of these genetic variants.

A powerful use of our database is in the study of the relationship between genotype and phenotype. A key challenge in complex trait genetics is how to identify the sequence variations that underlie complex traits. In addition to the phenotypic information and gene expression data on inbred strains provided by the CC, the aligned sequence of the 17 mouse genomes and the associated catalog of variants in transcriptomes will serve as a basic tool for understanding trait differences. Collectively, our presented database will allow further insights into the nature of functional variants and will help examine the path from genotype to phenotype.

Epigenomic approaches have recently been studied genome wide to tackle unanswered questions in human diseases, including cancer. It is known that enhancers in the human and mouse genomes are associated with active chromatin marks in a cell type–specific manner, whereas promoter and insulator elements tend to be ubiquitously occupied in multiple cell lines (18). During histone modifications, both lysine and arginine residues may be methylated. Methylated lysines are the best understood marks of the histone code, as specific methylated lysine marks match well with gene expression states. Methylation of lysines H3K4 and H3K36 is correlated with transcriptional activation, while demethylation of H3K4 is correlated with silencing of the genomic region. Methylation of lysines H3K9 and H3K27 is correlated with transcriptional repression (33, 34). The ECCO database enables us to map the three chromatin modification marks, histone H3 lysine 4 trimethylation (H3K4me3), histone H3 lysine 4 monomethylation (H3K4me1) and H3 lysine 27 acetylation (H3K27ac), in 13 adult tissues, 4 embryonic tissues and 2 primary cell lines for all the 17 mouse genomes. Using a similar approach, our method can also be applied to human ENCODE databases to connect genetics to epigenetics and to investigate further DNA methylation.

Many complex diseases result from gene–gene and gene–environment interactions that are not effectively modeled by isolated studies on fixed genetic backgrounds in mouse. The large panel of inbred CC mouse lines derived from eight genetically diverse strains captures almost 90% of the known variation present in laboratory mice (23), so it can provide better models for the genetic variation found in the human population. After 10 years of development, the CC project is now available for researchers. Research programs using the CC can be combined with our ECCO database for determining the molecular basis of complex diseases. For example, QTLs of complex phenotypes found by genetic mapping analysis can be further investigated using the ECCO web services. Polymorphisms associated with variable *cis*-regulatory elements in the target region can be considered as candidates and tested for biological variation that could determine the phenotypes of interest.

## Supplementary Data

Supplementary data are available at *Database* Online.
